# Post-Bariatric Surgery Changes in Quinolinic and Xanthurenic Acid Concentrations Are Associated with Glucose Homeostasis

**DOI:** 10.1371/journal.pone.0158051

**Published:** 2016-06-21

**Authors:** Marie Favennec, Benjamin Hennart, Marie Verbanck, Marie Pigeyre, Robert Caiazzo, Violeta Raverdy, Hélène Verkindt, Audrey Leloire, Gilles J. Guillemin, Loïc Yengo, Delphine Allorge, Philippe Froguel, François Pattou, Odile Poulain-Godefroy

**Affiliations:** 1 Univ. Lille, UMR 8199 - EGID, F-59000 Lille, France; 2 CNRS, UMR 8199, F-59000 Lille, France; 3 Institut Pasteur de Lille, F-59000 Lille, France; 4 Univ. Lille, EA 4483 - IMPECS - IMPact de l’Environnement Chimique sur la Santé humaine, F-59000 Lille, France; 5 CHU Lille, Service de Toxicologie-Génopathies, F-59000 Lille, France; 6 Univ. Lille, U1190 - EGID, F-59000 Lille, France; 7 Inserm, U1190, F-59000 Lille, France; 8 CHU Lille, Chirurgie générale et endocrinienne, F-59000 Lille, France; 9 Neuroinflammation group, Macquarie University, Sydney, NSW, 2109 Australia; 10 Department of Genomics of Common Disease, School of Public Health, Imperial College London, London, United Kingdom; GDC, GERMANY

## Abstract

**Background:**

An increase of plasma kynurenine concentrations, potentially bioactive metabolites of tryptophan, was found in subjects with obesity, resulting from low-grade inflammation of the white adipose tissue. Bariatric surgery decreases low-grade inflammation associated with obesity and improves glucose control.

**Objective:**

Our goal was to determine the concentrations of all kynurenine metabolites after bariatric surgery and whether they were correlated with glucose control improvement.

**Design:**

Kynurenine metabolite concentrations, analysed by liquid or gas chromatography coupled with tandem mass spectrometry, circulating inflammatory markers, metabolic traits, and BMI were measured before and one year after bariatric surgery in 44 normoglycemic and 47 diabetic women with obesity. Associations between changes in kynurenine metabolites concentrations and in glucose control and metabolic traits were analysed between baseline and twelve months after surgery.

**Results:**

Tryptophan and kynurenine metabolite concentrations were significantly decreased one year after bariatric surgery and were correlated with the decrease of the usCRP in both groups. Among all the kynurenine metabolites evaluated, only quinolinic acid and xanthurenic acid were significantly associated with glucose control improvement. The one year delta of quinolinic acid concentrations was negatively associated with the delta of fasting glucose (p = 0.019) and HbA1c (p = 0.014), whereas the delta of xanthurenic acid was positively associated with the delta of insulin sensitivity index (p = 0.0018).

**Conclusion:**

Bariatric surgery has induced a global down-regulation of kynurenine metabolites, associated with weight loss. Our results suggest that, since kynurenine monoxygenase diverts the kynurenine pathway toward the synthesis of xanthurenic acid, its inhibition may also contribute to glucose homeostasis.

## Introduction

**Fig 1:**
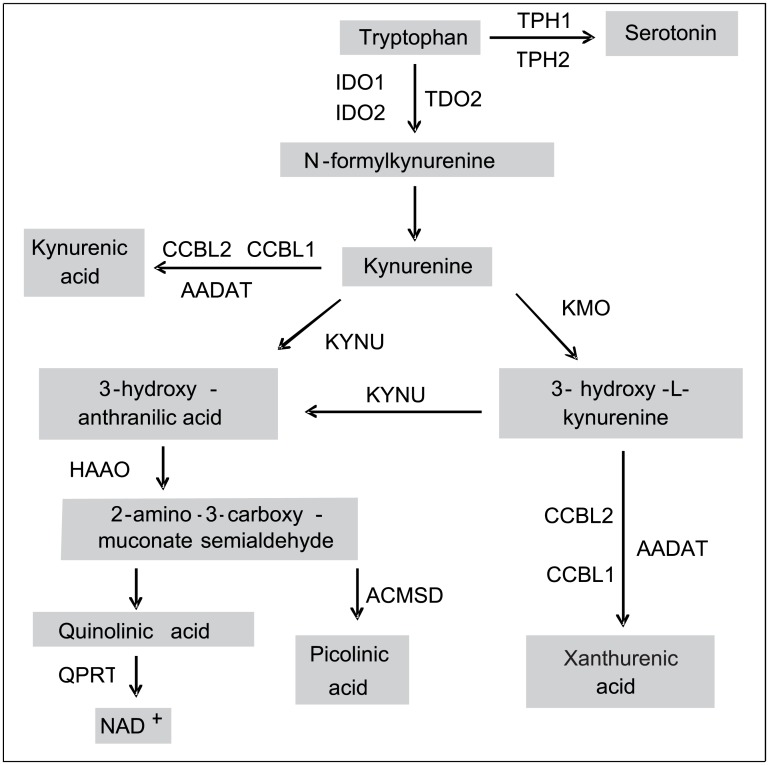
Schematic representation of the kynurenine pathway. IDO1, indoleamine 2,3-dioxygenase 1; IDO2, indoleamine 2,3-dioxygenase 2; TDO2, tryptophan 2,3-dioxygenase; TPH1, Tryptophan hydroxylase 1; TPH2, Tryptophan hydroxylase 2; AFMID, arylformamidase; KMO, kynurenine 3-monooxygenase; CCBL1, kynurenine aminotransferase I; AADAT, kynurenine aminotransferase II; CCBL2, kynurenine aminotransferase III; KYNU, kynureninase; HAAO, 3-hydroxyanthranilate 3,4-dioxygenase; QPRT, quinolinate phosphoribosyl transferase; ACMSD, aminocarboxymuconate semialdehyde decarboxylase.

Obesity is often associated with systemic insulin resistance and low grade inflammation and an immune activation of the adipose tissue that contributes to the development of metabolic and vascular complications [1]. The inflammation state increases the activity of indoleamine 2,3-dioxygenase (IDO1) [2], a key enzyme that degrades the essential amino acid tryptophan into kynurenine and initiates the kynurenine pathway that generates several metabolites collectively called the “kynurenines” (Fig 1). Our group and others previously showed that obesity is associated with an increased expression of the *IDO1* gene in the human adipose tissue and with increased concentrations of circulating kynurenines and kynurenine to tryptophan (K/T) ratio reflecting the increased enzymatic activity of IDO1, that also correlates with the increased expression of inflammatory markers in the adipose tissue [3–6]. We have also shown that kynurenine concentrations were positively associated with BMI and the insulin resistance index HOMA2-IR in a French general population cohort [7]. Tryptophan and kynurenine concentrations have already been shown to be correlated with HOMA-IR and HOMA-beta scores in hepatitis C virus patients [8]. An association of kynurenine, kynurenic acid and quinolinic acid with BMI and HOMA-IR was also recently demonstrated in another cohort [9].

Gastric banding and Roux-en-Y bypass are common bariatric surgery procedures [10]. Bariatric surgery improves the inflammation of the adipose tissue and decreases the concentrations of circulating inflammatory markers [11]. However, the metabolic effect of bariatric surgery is not fully understood and many patients may remain diabetic or insulin resistant after surgery despite significant weight loss through gastric banding [12].

Our objective was first to characterize the impact of surgical weight loss on the obesity-associated kynurenine pathway dysregulation and to determine whether kynurenines contribute to glucose homeostasis. In this study, we assessed the long term effects of weight loss on kynurenine pathway metabolite (KPm) concentrations and on metabolic traits in diabetic and normoglycemic women with severe obesity one year after bariatric surgery.

## Subjects and Methods

### Study design and characteristics of participants

**Fig 2:**
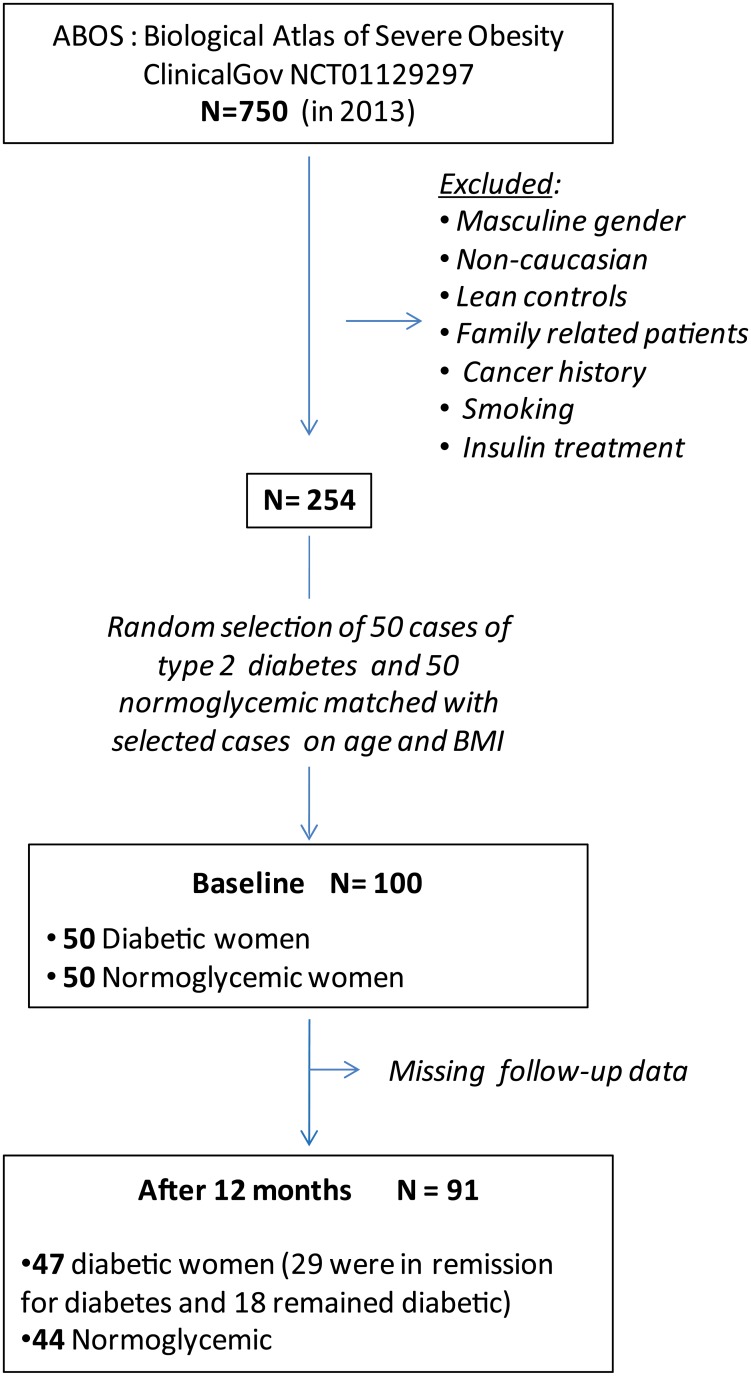
Flow chart of participants.

One hundred subjects enrolled in ABOS (Biological Atlas of Severe Obesity, ClinicalGov NCT01129297) were included in a previous study which evaluated circulating kynurenines in obese women [7]. The present work aims at determining tryptophan and kynurenine metabolite concentrations one year after bariatric surgery. After one year follow-up, data for ninety one participants were available (Fig 2). The selection criteria were previously described [7]. Briefly, since circulating tryptophan and kynurenine metabolite concentrations were shown to be significantly different between men and women [13] and in order to avoid gender effect, we studied only women, which are more prone to undergo bariatric surgery. Participants had severe obesity (BMI = 46,3 ±6,3 kg/m^2^) and met the criteria for bariatric surgery, as previously described [6]. We excluded non-Caucasian patients, family-related patients, smoking patients, patients with cancer history or insulin treatment for diabetes. Among the 47 diabetic patients, 39 were given an anti-diabetic therapy at inclusion; only 17 out of those 39 treated patients still had anti-diabetic therapy one year after surgery.

Written informed consent was obtained and the experimental design was approved by the Ethics Committee of the University Hospital of Lille, France. Clinical and biological features were assessed prospectively, first preoperatively and then 1 year after surgery. Relevant clinical and metabolic characteristics of the 91 participants are described in Table 1. Women were classified into two groups. According to the American Association of Diabetes, the oral glucose tolerance test criteria for normal glycaemia are fasting glucose concentrations (FG) < 5.5 mmol/L and 2-h glucose < 7.7 mmol/L and for type 2 diabetes: FG ≥ 7 mmol/L or 2-h glucose ≥ 11.1 mmol/L. At inclusion, cases with type 2 diabetes were matched with normoglycemic controls on age and BMI. Patients with missing follow-up data were excluded from the final analysis. Overall, 47 women were diabetic and 44 were normoglycemic at inclusion and no significant difference for age and BMI between the two groups of remaining patients was observed (Table 1). The choice between laparoscopic adjustable gastric banding (AGB) [12] or Roux-en-Y gastric bypass (RYGB) [14] was left to the surgical candidates after having been informed about the risks and benefits of each procedure according to contemporary knowledge. 24 individuals (16 normoglycemic and 8 diabetic) chose AGB and 67 individuals (28 normoglycemic and 39 diabetic) chose RYGB. The homeostasis model assessment (HOMA) was used as an index of insulin sensibility (HOMA2-S) and β-cell function (HOMA2-B) and the product of HOMA2-B and HOMA2-S was used as an index of β-cell compensatory capacity [15]. HOMA2-B and HOMA2-S were calculated using the HOMA calculator (https://www.dtu.ox.ac.uk/homacalculator/).

**Table 1:** Clinical characteristics of diabetic (DB) or normoglycemic (NG) women with obesity from the ABOS cohort at inclusion (M0) and 12 months after surgery (M12).

	M0		M12	
	DB (n = 47)	NG (n = 44)	DB (n = 47)	NG (n = 44)
Age, *years*	47.2 ± 5.8	44.9 ± 7.1	48.2 ± 5.8	45.9 ± 7.1
Weight, *kg*	125.3 ± 18.4	126.3 ± 20.2	91.3 ± 17.2	94±18.0
Weight loss, *%*	NA	NA	26.9 ± 11.4 †	25.0 ± 12.4 †
BMI, *kg/m*^*2*^	47 ± 6.1	45.5 ± 6.4	34.2 ± 5.9 †	34.0 ± 6.7 †
usCRP, *mg/L*	7.2 ± 3.1	6.9 ± 3.3	2.6 ± 2.8 †	3.3 ± 3.3 †
HbA1c, *%*	7.2 ± 1.4	5.8 ± 0.3*	5.9 ± 0.7 †	5.6 ± 0.3* †
Fasting glucose, *mmol/L*	8.1 ± 2.9	5.4 ± 0.4*	5.6 ± 1.7 †	4.9 ± 0.5* †
2H glucose, *mmol/L*	12.6 ± 4.6	6.0 ± 1.2*	5.8 ± 3.6 †	5.2 ± 1.8 †
Fasting insulin, *pmol/L*	100.1 ± 58.9	81.2 ± 36.4	56.2 ± 81.0 †	41.1 ± 25.0 †
HOMA2-S, *%*	84.0 ± 149.1	79.7 ± 37.8	176.4 ± 118.6 †	179.2 ± 104.7 †
HOMA2-B, *%*	69.0 ± 44.8	108.2 ± 34.0*	70.1 ± 28.5	78.5 ± 26.3 †
HOMA2-BS, %	38.6 ± 23.0	76.5 ± 19.0*	104.9 ± 53.8 †	121.6 ± 44.8 †
Surgery (AGB/RYGB)	7/40	15/29	7/40	15/29
Remission (Yes/No)	NA	NA	29/18	NA

Data are presented as mean ± SD,

* p < 0.05 t-test was used for statistical analyses between diabetic and normoglycemic women at M0 and M12.

^†^ p < 0.05 paired t-test was used for statistical analyses between baseline and at 12 months after surgery.

usCRP, ultrasensitive C Reactive Protein;

AGB, adjustable gastric banding;

RYGB, Roux-y-Gastric Bypass

### Determination of serum tryptophan and kynurenine concentrations

Fasting serum samples were collected before and 12 months after surgery. Paired samples of the same individual were analysed simultaneously. An analytical protocol based on liquid chromatography coupled with tandem mass spectrometry (LC-MS/MS) was used to measure tryptophan, serotonin, kynurenine, kynurenic acid, xanthurenic acid, 3-hydroxy-L-kynurenine and 3-hydroxy-anthranilic acid, as previously described [7]. Briefly, protein precipitation was performed with acetonitrile, containing tryptophane-D5 (50μM, CDN isotopes, Pointe-Claire, Canada) as an internal standard, and samples were injected onto an UPLC-MS/MS system (Acquity TQ Detector, Waters, Milford, MA) equipped with a HSS C18 column. Ions of each analysed compound were detected in a positive ion mode using multiple reaction monitoring. Picolinic acid and quinolinic acid were simultaneously measured using gas chromatography-tandem mass spectrometry (GC-MS/MS), as previously described [7]. Briefly, samples were mixed with internal standards (QUIN-D3 and PIC-D4, 2 μM) and methanol for protein precipitation. Samples were dried and then heated to allow derivatization with pentafluoropropionic anhydride (PFPA, Sigma Aldrich, Saint-Quentin Fallavier, France) and hexafluoroisopropanol (HFIP, Sigma Aldrich). The obtained derivatives were dried and dissolved in iso-octane and finally injected onto the GC-MS/MS equipment (Agilent 7890 coupled to Waters Quattro Micro GC, RAB293). Chromalinks software (Waters^™^) was used for data acquisition and processing.

### Statistical analyses

Statistical Mann-Whitney test for non normal distribution, T-test, paired T-test and Spearman correlation test were performed with the GraphPad Prism version 5.00 for Windows (GraphPad Software, San Diego, CA). The statistical tests are described with each result; the threshold of significance was set to p< 0.05.

A linear mixed model was used to assess the association between KPm concentrations and the concentrations of ultrasensitive C-reactive protein (usCRP), an inflammatory marker. In a second time, we used a multivariate linear regression model adjusted for weight loss to assess the association between the metabolic trait variations and tryptophan, serotonin and KPm concentration changes. In this study, since the weight loss and the type of bariatric surgery are confounding factors (T-test, p = 1.59x10^-13^), we chose to adjust our results for weight loss, knowing that we were simultaneously adjusting for the effect of the type of bariatric surgery. Variations after surgery were evaluated by the difference between concentrations at one year and concentrations at baseline ([M12]–[M0]); for readability purposes, we will refer to this difference as delta (Δ). The analysed metabolic traits were fasting plasma glucose, fasting plasma insulin, glycated hemoglobin A1c (HbA1c), the homeostasis model assessment of β-cell function (HOMA2-B) and of insulin sensitivity (HOMA2-S) and their product called HOMA2-BS. We used a Benjamini-Hochberg correction for multiple testing; the threshold of significance for corrected p-values was set to p < 0.05. Statistical analyses were performed using R version 3.0 (packages nlme and multtest).

## Results

At baseline, age and BMI were not significantly different between the diabetic (N = 47) and normoglycemic (N = 44) groups (Table 1). Mean BMI significantly decreased one year after bariatric surgery in both groups (p < 0.001) (Table 1). We observed a 25% decrease of the initial body weight in the group of normoglycemic women *vs* 26.9% in diabetic women (not significant). As expected, fasting glucose and insulin concentrations, HOMA2-S, HOMA2-BS and usCRP concentrations were significantly improved twelve months after bariatric surgery in both groups (Table 1). We defined the rate of diabetes remission at one year by the absence of diabetes-specific treatment and fasting blood glucose concentrations < 6.1 mmol/L [16]. Among the 47 diabetic women at baseline, 29 were in remission 12 months after surgery (62%). The rate of diabetes remission is different between the two types of intervention and is of 50% (4/8) for AGB and of 64% (25/39) for RYGB, but this is not significant which is certainly due to the small sample size since only 8 out of the 47 diabetic patients chose AGB.

**Fig 3:**
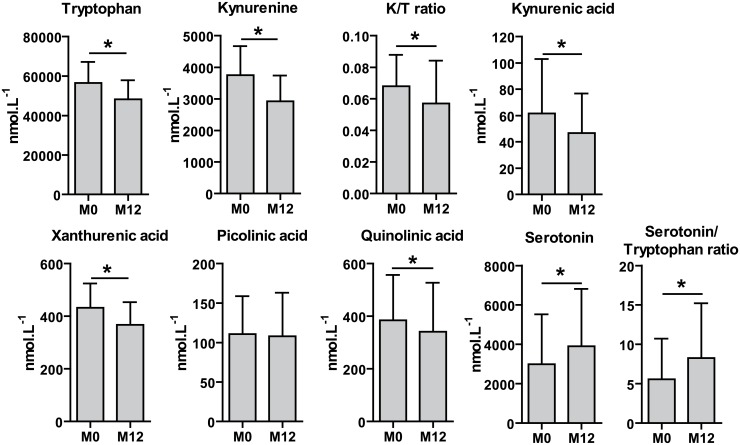
Tryptophan, serotonin, KPm concentrations, K/T ratio and serotonin over tryptophan ratio in the serum of women with obesity at inclusion and 12 months after bariatric surgery. Data are presented as mean ± SD; Mann-Whitney test was used for statistical analyses, * statistically significant at 0.05 after a Benjamini-Hochberg correction for multiple testing.

One year after surgery, tryptophan, kynurenine, kynurenic acid, xanthurenic acid and quinolinic acid concentrations significantly decreased in combined groups (e.g. 91 patients analysed simultaneously; p = 2.2x10^-10^, p = 2.7x10^-4^, p = 1.3x10^-4^, p = 1.6x10^-7^ and p = 8.4x10^-5^ respectively) (Fig 3) or in each glycemic group analysed independently (data not shown). Among the KPm which were analysed, only picolinic acid concentrations did not decrease after the surgery. Conversely, serotonin concentrations and the ratio of serotonin over tryptophan increased 12 months after bariatric surgery (p = 2.1x10^-5^ and p = 1.3x10^-5^, respectively). Linear mixed model analysis showed that tryptophan concentrations were negatively correlated with C Reactive Protein concentrations (usCRP, β = -0.018; p = 0.0072) and that kynurenine and kynurenic acid concentrations were positively correlated with usCRP concentrations (β = 0.019; p = 0.028 and β = 0.038; p = 4.21x10^-2^, respectively) (Table 2). The K/T ratio decreased after bariatric surgery (p = 7.5x10^-4^) and was also positively correlated with usCRP concentrations (β = 0.036; p = 1.1x10^-4^). In addition, we observed that serotonin and the ratio of serotonin over tryptophan were negatively correlated with usCRP concentrations (β = -0.055; p = 0.014; β = -0.069; p = 0.0032, respectively).

**Table 2:** Association between usCRP and serum concentration of tryptophan, kynurenine, kynurenic acid, serotonin, K/T ratio and serotonin over tryptophan ratio.

	usCRP, *mg/L*	
	β	p-value
Tryptophan, *mmol/L*	**-0.018**	**7.2x10**^**-3**^
Kynurenine, *mmol/L*	**0.019**	**0.028**
Kynurenic acid, *mmol/L*	0.038	0.042
K/T ratio	**0.036**	**1.1x10**^**-4**^
Xanthurenic acid, *mmol/*	-0.014	0.083
Quinolinic acid, *mmol/L*	0.021	0.067
Serotonin, *mmol/L*	**-0.055**	**0.014**
Serotonin / tryptophan ratio	**-0.069**	**3.2x10**^**-3**^

usCRP, ultrasensitive C Reactive Protein

β coefficients from linear mixed models are shown and correlations in bold are statistically significant at 0.05 after a Benjamini-Hochberg correction for multiple testing.

As it was demonstrated that metformin, the first-line medication for the treatment of type 2 diabetes, could inhibit TDO2, a key enzyme in tryptophan metabolism [17], we assessed the potential impact of diabetes therapy (except insulin, as patients on insulin therapy were excluded) on KPm concentrations. No association was demonstrated between any KPm concentration and the presence of metformin therapy, alone or together with other anti-diabetic drug, either at inclusion or one year after surgery (data not shown).

KPm concentrations were not significantly different between the normoglycemic and diabetic groups, neither at inclusion, nor one year after surgery (Table 3). Women were then reclassified according to their diabetic status 12 months after surgery and regrouped into “diabetic12m” and “nondiabetic12m” categories. No significant differences in any KPm concentrations was observed between the two reclassified groups (Table 3).

**Table 3:** Association between diabetic status and serum concentration of metabolites and K/T ratio, at inclusion and one year after surgery. One year after surgery, analysis was performed first according to women diabetic status at inclusion and then according to their diabetic status after reclassification.

	At inclusion		One year after surgery		One year after surgery (reclassified)	
	β	p-value	β	p-value	β	p-value
Tryptophan, mmol/L	0.0587	0.2943	0.0348	0.6041	-0.0354	0.6264
Kynurenine, mmol/L	0.0364	0.6227	-0.0522	0.5494	-0.0639	0.4982
Kynurenic acid, mmol/L	0.1018	0.6325	0.1636	0.4288	-0.3710	0.0879
K/T ratio	-0.0224	0.7810	-0.0870	0.3754	-0.0285	0.7888
Xanthurenic acid, mmol/L	0.0482	0.4862	-0.0232	0.7516	0.0090	0.9094
Quinolinic acid, mmol/L	0.2203	0.0991	0.0800	0.5608	-0.2204	0.1367
Serotonin, mmol/L	-0.2045	0.5564	-0.1333	0.6220	0.1438	0.6259

Patients with RYGB had a higher decrease of the initial body weight (30.75% in average) than patients with AGB (12.64% in average) after one year (p-value of 1.59x10^-13^). In this study, weight loss and the type of bariatric surgery are confounding factors. We have chosen to display only the results after adjusting for weight loss in this study after assessing that associations obtained after adjusting for weight loss, for intervention or for both are globally equivalent (data not shown). Associations between KPm and quantitative metabolic trait changes after surgery were analysed after adjusting for weight loss. Quinolinic acid and xanthurenic acid concentrations changes with time (Δ) were correlated with the Δ of the analysed quantitative metabolic traits. Indeed, the Δ quinolinic acid was negatively correlated with Δ fasting glucose (β = -1.311; p = 0.019) and with Δ HbA1c (β = -0.678; p = 0.014), and, conversely, the Δ xanthurenic acid was negatively correlated with the Δ HOMA2-S (β = -113.4; p = 0.0018) and Δ HOMA2-BS (β = -38.69; p = 0.0026) indexes (Table 4). As expected, the Δ quinolinic acid over xanthurenic acid ratio was negatively correlated with the Δ fasting glucose (β = -1.098; p = 0.0075) and positively correlated with the improvement of the HOMA2-S (β = 91.12; p = 0.00074) and HOMA2-BS (β = 27.09; p = 0.00082) indexes (Table 4).

**Table 4:** Correlations between metabolic trait changes and KPm level changes after surgery.

	Δ Quinolinic acid		Δ Xanthurenic acid		Δ Quinolinic acid / xanthurenic acid	
	β	p-value	β	p-value	β	p-value
Δ Fasting glucose, *mmol/L*	**-1.311**	**0.019**	0.953	0.144	**-1.098**	**0.0075**
Δ Fasting insulin, *pmol/L*	-1.85	0.441	5.34	0.052	-3.15	0.0713
Δ HbA1c, *%*	**-0.678**	**0.014**	0.154	0.636	-0.431	0.035
Δ HOMA2-B, *%*	6.97	0.485	8.37	0.469	0.391	0.957
Δ HOMA2-S, *%*	68.92	0.0667	**-133.4**	**0.0018**	**91.12**	**0.00074**
Δ HOMA2-BS, %	21.22	0.059	**-38.69**	**0.0026**	**27.09**	**0.00082**

The difference between the levels at 12 months and at inclusion ([M12]–[M0]) was used to analyse the changes in metabolic traits and KPm levels. For readability purposes, we refer to this difference as delta (Δ).

β coefficients from multiple linear model are shown adjusted for weight loss and correlations in bold are statistically significant at 0.05 after a Benjamini-Hochberg correction for multiple testing

## Discussion

Our study shows that the K/T ratio significantly decreases after bariatric surgery-induced weight loss. A previous study performed on 22 individuals did not demonstrate any significant decrease of the K/T ratio after bariatric surgery [3]. Recently, a decrease in tryptophan and kynurenine concentrations was shown in 38 individuals after a caloric restriction-induced weight loss, but the K/T ratio was not significantly modified [18]. These contradictory results may be due to the small sample size and the insufficient statistical power of these previous studies. Our study revealed that tryptophan and KPm concentrations significantly decreased one year after bariatric surgery and were correlated with the decrease of the well-known marker of systemic inflammation usCRP. We hypothesized that in patients with obesity, K/T ratio is mainly driven by IDO1 activity. However, we could not exclude that tryptophan might be converted into kynurenine by cortisol-inducible TDO2 enzyme as well [19]. These results are in line with our previous observations that kynurenine concentrations are higher in individuals with obesity compared to lean patients [6] and that kynurenine and several other KPms are positively correlated with BMI in women from the same cohort [7]. However, among the KPms, picolinic acid concentrations did not decrease at 12 months after surgery, suggesting that the synthesis of picolinic acid is not affected by weight loss. Accordingly, we previously showed that the enzyme which catalyzes the synthesis of picolinic acid is not expressed in the human adipose tissue [7] which may explain our current data obtained after adiposity reduction.

Our results also revealed that serotonin synthesis increased one year after surgery, suggesting that in this new energetic state tryptophan metabolism is derived preferably towards serotonin rather than kynurenine synthesis. In the same way, it has been suggested that the number of serotonin-expressing enteroendocrine cells increases in the rat after bariatric surgery [20]. However, a limitation of our study is that we evaluated serotonin concentrations in the serum which is less accurate than in platelets as there is a risk of platelet-derived serotonin contamination during the preparation of serum [21].

The presumed mechanisms of action of RYGB and of AGB are multiple and thought to be of different nature [22]. Malabsorption of nutriments, as well as dramatic changes in diet observed after surgery, are susceptible to impair tryptophan concentrations and it was shown that other amino acid profiles were also modified after bariatric surgery [23]. In order to properly assess the effect of the type of bariatric surgery, we would have to study for instance patients after comparable weight loss, independently of the delay after surgery. In our study, after adjusting for post-surgery weight loss, we found a remaining significant association between several KPm concentration changes and the improvement of glucose homeostasis, but no significant association between diabetes remission and KPm concentrations. Indeed, the ratio of quinolinic acid over xanthurenic acid is positively associated with better insulin sensitivity and glucose control.

The enzyme kynurenine 3-monooxygenase (KMO) is located at a critical branching point in the kynurenine pathway such that the inhibition of this enzyme by either pharmacological or genetic means may shift the flux towards the formation of kynurenic acid rather than to the synthesis of xanthurenic acid. The inhibition of KMO has suggested that KMO regulates the production of downstream KP metabolites. We have previously shown that the adipose tissue expression of KMO was associated with higher HbA1c in women with obesity and was expressed preferentially in pro-inflammatory M1 macrophages [7]. Both the activation of KMO and, consequently, the potential synthesis of xanthurenic acid may be deleterious to glucose homeostasis. Vitamin B6 deficiency was shown to lead to a large increase in the generation of xanthurenic acid [24]. Interestingly, vitamin B6 deficiency was demonstrated in obesity [25]. Patients undergoing bariatric surgery were sporadically supplemented in vitamins after the intervention to avoid any deficiency; unfortunately we had no information on vitamin B6 concentrations in patients’ sera. The compliance to vitamin B6 supplementation may also be a factor modulating the levels of xanthurenic acid.

The relationship between kynurenine metabolites and type 2 diabetes, especially xanthurenic acid, has been suspected for a long time [26–27] and these observations were recommended to be further investigated [28]. Xanthurenic acid has been described as a zinc chelator and may interfere with insulin secretion [29]. More recently, some studies suggested the involvement of KPm in the development of insulin resistance [8] and the increase of xanthurenic acid concentration in type 2 diabetes [30]. However, regarding the small number and heterogeneity of the patients (from both genders, older diabetic patients than control patients), the results of these studies require confirmation. Moreover, it was clearly demonstrated in a huge cohort (n = 7052) that age and gender are modulating factors of KPm concentrations [13].

Considering our data, although we observed no difference in KPm concentrations between the diabetic and normoglycemic groups, after weight loss adjustment, we found that a preferential decreased activation of the xanthurenic acid branch was associated with glucose control improvement one year after bariatric surgery.

In conclusion, we showed that tryptophan and KPm concentrations decreased after a bariatric surgery in women with obesity, and our results suggest potential interactions between kynurenine metabolite production, inflammation and glucose metabolism.
